# DNA Methylation: Genomewide Distribution, Regulatory Mechanism and Therapy Target

**DOI:** 10.32607/actanaturae.11822

**Published:** 2022

**Authors:** D. S. Kaplun, D. N. Kaluzhny, E. B. Prokhortchouk, S. V. Zhenilo

**Affiliations:** Institute of Bioengineering, Research Center of Biotechnology, Russian Academy of Sciences, Moscow, 119071 Russia; Institute of Gene Biology, Russian Academy of Sciences, Moscow, 119071 Russia; Engelhardt Institute of Molecular Biology, Russian Academy of Sciences, Moscow, 119991 Russia

**Keywords:** DNA methylation, DNA methyltransferases, G-quadruplexes, TET dioxydenases, methyl-DNA binding proteins

## Abstract

DNA methylation is the most important epigenetic modification involved in the regulation of transcription, imprinting, establishment of X-inactivation, and the formation of a chromatin structure. DNA methylation in the genome is often associated with transcriptional repression and the formation of closed heterochromatin. However, the results of genome-wide studies of the DNA methylation pattern and transcriptional activity of genes have nudged us toward reconsidering this paradigm, since the promoters of many genes remain active despite their methylation. The differences in the DNA methylation distribution in normal and pathological conditions allow us to consider methylation as a diagnostic marker or a therapy target. In this regard, the need to investigate the factors affecting DNA methylation and those involved in its interpretation becomes pressing. Recently, a large number of protein factors have been uncovered, whose ability to bind to DNA depends on their methylation. Many of these proteins act not only as transcriptional activators or repressors, but also affect the level of DNA methylation. These factors are considered potential therapeutic targets for the treatment of diseases resulting from either a change in DNA methylation or a change in the interpretation of its methylation level. In addition to protein factors, a secondary DNA structure can also affect its methylation and can be considered as a therapy target. In this review, the latest research into the DNA methylation landscape in the genome has been summarized to discuss why some DNA regions avoid methylation and what factors can affect its level or interpretation and, therefore, can be considered a therapy target.

## INTRODUCTION

**Fig. 1:**
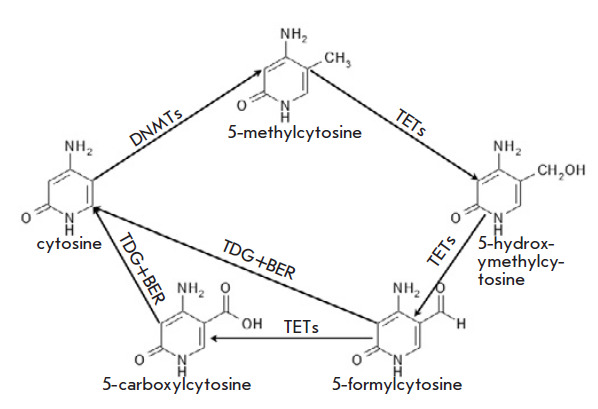
Cytosine methylation and demethylation scheme

Cytosine is referred to as the fifth DNA base, and cytosine residue methylation is the most common DNA modification in mammalian cells. Cytosine residues in CpG dinucleotides are most often subject to methylation. However, the methylated cytosines outside CpG dinucleotides may account for 25–50% of all mC in stem cells and neurons [1]. In mammals, about 70–80% of cytosines in CpG dinucleotides are methylated [2]. De novo DNA methylation is catalyzed by the DNMT3a/3b DNA methylatransferases responsible for methylation in different genome regions and that are not interchangeable [3, 4]. DNA methylation during replication is maintained by DNMT1 DNA methyltransferase. DNA demethylation occurs both passively, during cell division, and actively, due to enzyme activity. The key factors involved in active demethylation are TET1,2,3 dioxygenases. TET proteins oxidize methylcytosine to hydroxymethylcytosine and, then, formylcytosine and carboxycytosine, which then produce cytosine as a result of excision repair by thymine-DNA glycosylase (TDG/NEIL) (Fig. 1) [5]. Methylcytosine derivatives are not only considered as intermediate states between methylated and non-methylated bases, but also as DNA modifications affecting the binding of transcription factors, as they are involved in gene expression regulation (Methylcytosine derivatives are discussed in survey [6]).

The key changes in DNA methylation during the organism’s development are associated with cell differentiation. Differentiated cells are typically characterized by stable DNA methylation patterns, which can still vary due to external stimuli, various pathological processes, and ageing [7, 8, 9, 10, 11]. Dynamic DNA methylation changes in differentiated cells are also observed during memory formation and training in neural cells [12, 13]. DNA methylation in differentiated cells turns out to be stable in the remaining cases. Thus, DNA methylation can be considered as a target for therapy and the diagnostics of various pathogenetic conditions based on DNA methylation abnormalities affecting gene transcription.

The key features of DNA methylation distribution in the genome are presented in this survey. The factors affecting DNA methylation onset, maintenance, and demethylation are analyzed based on recently published data. The possibility for therapeutic use of these factors is discussed.

## 1. THE DNA METHYLATION DISTRIBUTION PATTERN IN MAMMALIAN CELLS

**Fig. 2:**
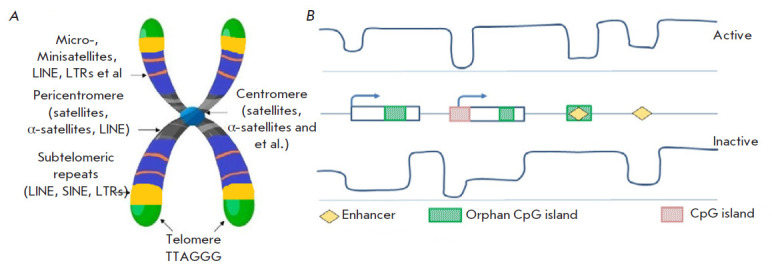
Landscape of the DNA repetitive sequences. (*A*) Location of various repeating sequences on a chromosome. (*B*) DNA methylation profile in the genome, depending on the activity of promoters and enhancers and the presence of CpG islands

About 90% of all methylated CpG sistes in mammalian genomes are located in various repeating sequences, such as satellite repeats and mobile elements [14]. The largest number of CpG-rich repeating elements are found in structural chromosomal regions: centromere, pericentromere, and subtelomere (Fig. 2A). Genome-wide nonopore sequencing in humans has made it possible to not only read the sequences of repeating elements, but also to analyze their methylation in the genome: so, a significant degree of methylation has been observed under normal conditions [2, 15]. It is of note that methylation of the duplicated/repeating sequences located in various chromosomal regions may differ significantly [2]; i.e., a specific methylation pattern of repeating sequences is not only determined by the sequence repeating it self, but by its chromosomal surroundings as well. Hypomethylation of various repeating elements is characteristic of various pathological conditions, including oncogenesis, immunodeficiency, as well as autoimmune, neurological, and mental disorders [7, 8, 9, 16, 17]. The necessity of satellite repeat methylation in centromere and pericentromere regions is associated with the correctness of chromosome disjunction in replication [18]. In contrast, methylation of mobile elements, transposons, and retrotransposons is aimed at suppressing their transcription. Demethylation of these repeats results in their active transcription and transposition, which fosters genome instability. It is possible that this is a redundant mechanism, because early transposons and retrotransposons are typically characterized by mutations and deletions in the sequences coding for transposase, which leads to inactive protein formation.

The mammalian genome includes CpG dinucleotides that avoid methylation. These CpG sites are usually included in the so-called CpG islands that are DNA regions where the GC pair content exceeds 50%, while the expected-to-observed CpG content is above 0.6. About 60% of the promoters include CpG islands. Lysine 4 residue trimethylation in the histone H3 molecule (H3K4me3) is an active chromatin modification typical for these regions, regardless of promoter activity [19]. Active chromatin is a DNA region where histone modifications, such as acetylation and H3K4me3, lead to DNA accessibility for transcription activators. The presence of H3K4me3 in the promoter regions of inactive genes facilitates transcription initiation but not mRNA synthesis. Meanwhile, there is a series of inactive gene promoters, including non-methylated CpG islands, where H3K4me3 is not detected. The genes located in clusters with three or more homologous genes coding for olfactory receptors, keratins, apolipoproteins, interleukins, and leukocyte antigens are most commonly attributed to this class [19]. Methylation of CpG islands in the promoter regions correlates with transcription suppression and may occur both under normal (e.g., during organism development) and pathological conditions [20]. For instance, malignant cell transformation and metastasis are typically characterized by hypermethylation of CpG islands in the promoters of oncosuppresor genes; i.e., the proteins involved in cellular adhesion and DNA repair. In most cases, such hypermethylation results in transcription suppression. However, it should be noted that promoter hypermethylation in tumors may occur in the genes considered transcriptionally inactive in the same tissue under normal conditions. In other words, their hypermethylation has no effect on expression suppression but, rather, reinforces their inactive status [21].

Promoters that include a small quantity of CpG dinucleotides are typical of tissue-specific genes and the genes involved in organism development. Methylation in these promoters does not always correlate with transcription suppression [22]. Inn a comparative analysis of brain and retinal cells, methylation of 66% of differentially methylated promoters correlated negatively with transcription. Thus, methylation in these promoters corresponds to transcription suppression. At the same time, promoter methylation was observed in 34% of transcriptionally active genes in [22].

The CpG islands that do not overlap with promoter regions are called orphan islands. The number of such islands is about half that of the promoter islands. Orphan islands often include H3K4me3 active chromatin modification and can initiate new transcripts [23]. Many orphan islands are subject to methylation during organism development, which makes them lose active chromatin modifications. Methylation in an orphan island inside a gene prevents the occurrence of transcription initiation sites inside the gene and correlates with active transcription [24]. Methylation inside genes may prevent Polycomb protein binding in the PRC2 repressor complex, which facilitates active transcription as well [25]. About 90% of orphan islands may act as tissue-specific enhancers [26]. The presence of a CpG island amplifies the enhancer’s regulatory activity [27]. Active enhancers that include orphan islands are hypomethylated, while the classical enhancers operating in all tissue types show variable methylation [27] (Fig. 2B).

Methylation maps created for the whole genomic DNA in various cell types and the information regulatory activity of the elements make it possible to consider DNA methylation as a tool for transcription activity regulation with regard to correction or identification of the various pathogenetic states associated with changes in DNA methylation.

## 2. DNA METHYLATION HOMEOSTASIS

DNA methylation homeostasis is based on a complex regulatory network that balances methylation and demethylation. The key mechanisms maintaining homeostasis in cellular proliferation and differentiation are as follows: 1) passive genome-wide demethylation and maintainance of the methylation pattern by DNMT1 during replication and 2) targeted de novo methylation and active demethylation in specific regions. The factors involved in homeostasis are discussed in the present Chapter.

**2.1. Maintaining the non-methylated state in DNA regions**

About 20% of CpG dinucleotides, most of them CpG islands, avoid methylation. The main factors preventing their methylation include histone modifications, DNA intercations with certain transcription factors (TF), and the DNA primary and secondary structure.

**Fig. 3:**
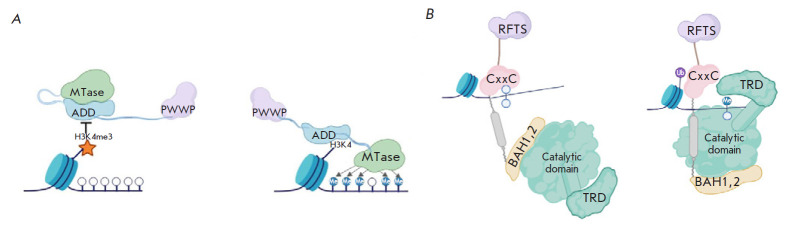
Binding scheme (*A*) DNMT3a/3b to unmodified H3K4, the presence of H3K4me3 prevents the ADD domain from binding to DNA, which leads to autoinhibition of the enzyme; (*B*) DNMT1 to DNA, the interaction with unmethylated DNA leads to inhibition of the catalytic domain [29, 30]

2.1.1. H3 lysine 4 trimethylation. The presence of trimethylated H3K4 is among the reasons explaining the stability of CpG islands against de novo methylation regardless of the transcriptional activity of the region. H3K4me3 prevents the attraction of de novo DNA methyltransferases DNMT3a/3b and their regulator, DNMT3L, showing no own catalytic activity to the DNA [28]. DNA methyltransferases DNMT3a/3b include a catalytic domain showing methyltransferase activity (MTase), as well as ADD and PWWP domains involved in chromatin binding. DNA methyltransferases, when in their DNA-unbound form, are inactive due to autoinhibition: the ADD domain interacts with the catalytic domain hindering its activity (Fig. 3). The ADD domain is unable to interact with H3K4me3. At the same time, non-modified H3K4 interacts with the ADD domain of DNMT3a/3b, thereby disrupting ADD binding to the catalytic domain and facilitating the manifestation of methyltransferase activity [28, 29]: Thus, DNA methylation and H3K4 methylation are mutually exclusive phenomena (Fig. 3A).

The case when DNA methylatransferase DNMT1 is maintained is different. DNMT1 is localized in promoter regions, including non-methylated CpG islands, and is not involved in their methylation. DNMT1 includes the following domains: RFTS (replication foci-targeting sequence), ZF-CxxC, two BAH- (bromo-adjacent homology) domains, and the catalytic domain. The CxxC domain of DNMT1 may bind to sequences including non-methylated CpG dinucleotides. Meanwhile, the BAH1 domain physically intervenes through the interaction between the catalytic domain and the DNA, thereby preventing de novo methylation (Fig. 3B) [30].

A particular feature of CpG islands is their ability to bind to TF and enzymes containing the ZF-CxxC domain (CFP1, MLL1/2, KDM2A/2B, TET1/TET3, DNMT1) [31]. Many of these factors are represented by or bind to the histone methyltransferases that modify H3K4, which hinders the attraction of DNA methyltransferases. It should be noted that the lower the gene promoter activity, the higher the need for H3K4me3 to maintain its non-methylated state [32, 33].

2.1.2. TET dioxygenases. TET dioxygenases (ten-eleven translocation) are the enzymes that oxidize methylcytosine for the subsequent excision repair. TET proteins are attracted to the DNA through various mechanisms. TET bind to the CpG islands by their CxxC domain or other transcription factors with a CxxC domain. TET proteins may also be attracted to DNA without the involvement of CpG islands, through messenger proteins, such as Klf4, Nanog, REST, GADD45, CEBPa, etc.; e.g., TET1 and TET2 are attracted to DNA by binding to the TF Nanog, leading to the demethylation of the regulatory gene regions involved in the maintenance of the pluripotent cellular state [34]. Notably, TET proteins, similar to many CxxC-containing proteins, affect H3K4 trimethylation. TET interact with OGT transferase (O-GLCNac transferase), which in turn forms a complex with SET1 and MLL histone methyltransferases trimethylating H3K4 [35].

The so-called pioneer factors play a major part in DNA demethylation by TET dioxygenases [36]. They interact with closed, inactive chromatin and change its accessibility for transcription activators. They show their peak activity during organism development, immune system maturation, oncogenesis, and somatic cell reprogramming. Pioneer factors include FOXA1, FOXO, Sox, Pax, GATA, Oct4, PU1, CEBPα, and other TF [37]. The key feature of these factors is their ability to recognize not just a DNA sequence, but a DNA region in a nucleosome context as well [38, 39]. This explains why DNA methylation is not always critical for pioneer factor attraction. In fact, many pioneer factors are methylation-insensitive or have recognition sites that do not contain CpG dinucleotides, which is illustrated by the cases of ASCL1 and FOXA1 [40, 41]. Nevertheless, the pioneer factors Oct4 and Klf4 interact both with sequences not containing CpG and sites containing CpG. In the latter case, Oct4 and Klf4 only bind to the methylated sites [42]. The pioneer factors capable of forming complexes with TET dioxygenases include Klf4, CEBPa, and TFCP2l1 [37]. The functional significance of TET2 interaction with Klf4 and CEBP in the process of somatic cell reprogramming has been demonstrated: e.g., the pioneer factors Klf4 and CEBPa attract TET2 dioxygenase to methylated enhancer sequences, which leads to their demethylation and activation [37]. Here, methylation decrease in certain chromatin regions, including in the Klf4 binding sites, is followed by chromatin remodeling. TET2 knockout cells are not subject to reprogramming [37]. Thus, DNA demethylation by TET enzymes is among the key stages of cellular reprogramming.

Despite the involvement of TET proteins in demethylation in many regions, their removal does not lead to catastrophic changes in the genome-wide DNA methylation level. The main DNA methylation changes in the case of TET knockout have to do with distal regulatory elements and enhancer sequences [43].

2.1.3. DNA secondary structure. Changes in conformation – aka DNA secondary structure – are among the factors contributing to the maintenance of a non-methylated state in CpG islands.

One of these factors is an R-loop, which is an RNA-DNA hybrid and a displaced DNA strand. GADD45A binding to an R-loop in the promoter of the tumor suppressor gene TCF21 attracts TET1, facilitating local demethylation in the region [44]. Thus, the DNA secondary structure may affect DNA demethylation by binding to TET dioxygenases.

**Fig. 4:**
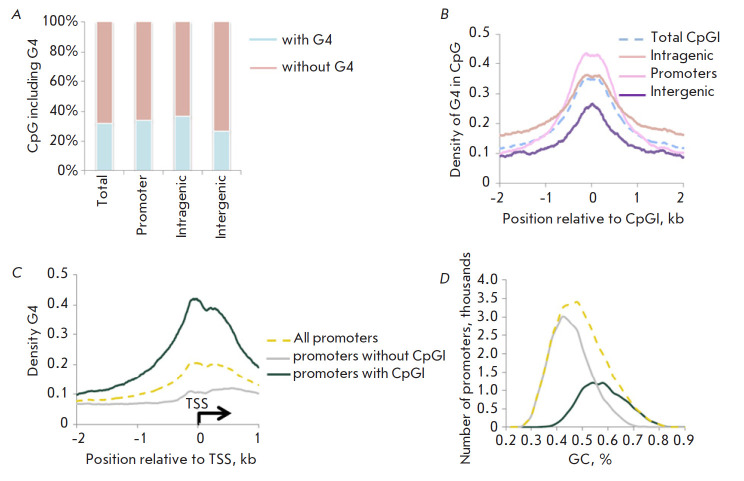
Distribution of potential G4 sequences in CpG islands. (*A*) The proportion of CpG islands with G4, (*B*) the distribution density of G4 near CpG islands depending on the localization in the genome. (*C*) G4 density and (*D*) GC composition in promoter regions depending on the presence of CpG islands

G-quadruplexes can also have an effect on the methylation of CpG islands and the CpG dinucleotides not included in the islands. It is an established fact that the GC-rich regulatory regions of eukaryotic genomes are capable of changing local DNA conformation by arranging themselves into alternative structures in the form of G-quadruplexes (G4) [45]. The secondary G-quadruplex (G4) structure is formed by guanine-rich sequences. The G-G base-paired Hoogsteen interaction results in guanine quartet formation, and stacks of such quartets stabilized by potassium cations form the G4 core. The thermodynamic stability of these structures depends on the nucleotide sequence and sometimes exceeds that of the DNA double helix. There are several theoretical and experimental approaches to determining potential G4 regions. Stable G4s formed in the genomic DNA in the presence of potassium ions act as the barrier for DNA polymerase. It often becomes an obstacle for PCR amplification in genome regions including GC-rich sites prone to the formation of G4 structures [46]. The approach based on high-performance sequencing of the errors occurring in the presence of potassium ions is currently considered the best in the experimental prediction of the G4 refolding potential in genomic DNA [47]. A change in the DNA conformation affects its physical and chemical properties and the affinity of various proteins specific to a certain nucleotide sequence. Methylation in the CpG context may change the energy barrier for a transition between the DNA double helix and non-canonical DNA structures, in particular G4 [48]. About 30% of CpG islands include nucleotide sequences capable of forming G4 structures (Fig. 4A). Intragenic CpG islands are relatively rich in quadruplex sequences, while the probability of their occurrence in intergenic CpG islands is low. The highest G4 density significantly above the average for all promoters (Fig. 4C) is detected in promoter CpG islands (Fig. 4B). The maximum G4 density is observed near the transcription start site (TSS). Decreasing G4 occurrence in promoter regions with no CpG islands may be related to the differences in the GC-contents between the promoters overlapping with CpG islands and those removed from them (Fig. 4D). The probability of encountering a potential G4-quadruplex depends significantly on the GC-content, even in a randomly generated nucleotide sequence. The probability of encountering a potential G4 in a random sequence with a GC-content of 40% is about one G4 per a million base pairs, while increasing the GC-content in a random sequence to 70% increases the G4 occurrence probability to one per one thousand base pairs [49]. The probability of encountering a G4 sequence in a higher organism genome is above average. These sequences may have an important regulatory role, which is somewhat confirmed by positive evolutionary selection [50]. The presence of G4 in promoters is often associated with transcription suppression [51]. Nevertheless, G4 in stem cells is detected in active promoters and the sites interacting with them; i.e., enhancers, superenhancers, and TF binding sites determining the cell type. In addition to active regulatory elements, G4s are found in regions with bivalent chromatin modifications; i.e., the ones containing both active and inactive chromatin modifications. A decrease in the detected G4 structures associated with cell differentiation correlates with the occurrence of a closed chromatin [48, 52]. Quadruplex structures may interact with DNA-methyltransferases DNMT1, DNMT3A, and DNMT3B in vitro [53, 54]. Indeed, non-methylated sequences in CpG islands containing quadruplexes are rich in DNMT1 binding sites. Meanwhile, interaction between DNMT1 and G4 leads to its DNA methyltransferase inactivation [53]. Thus, G4 formation hinders DNA methylation. This is confirmed by the correlation between the presence of stable quadruplexes in open chromatin and DNA hypomethylation. This correlation is primarily characteristic of sites with a low GC content. Relatively low methylation is also typical for CpG islands in a closed chromatin containing quadruplexes, compared to regions free of quadruplexes [55].

It is still unclear what DNMT1 activity – specifically the binding to non-methylated CpG sites, when domain positioning hinders the catalytic activity, or interactions with non-canonical DNA structures – is critical in maintaining the non-methylated status of CpG islands. Notably, there are genome regions where DNMT1 binding to DNA manifests de novo methyltransferase activity. These regions include LTR-retrotransposons enriched with H3K9me3 and TRIM28. Here, DNMT1 de novo activity is regulated by UHRF1 [56]. Thus, the presence of co-factors is also critical for the manifestation of DNMT1 de novo activity, in addition to domain positioning.

2.1.4. Competition between transcription factors and DNA methyltransferases. TF binding to DNA can occur with attraction of DNA methylatransferases, thereby protecting DNA from methylation. Sp1 is the classic example of this competition between TF and DNA methyltransferase binding. Sp1 interacts with the non-methylated sequences CCGCCC CpG islands are enriched with and intervenes, with attraction of DNA methyltransferase [57]. Mutation in an Sp1 binding site leads to its increased methylation and reduced transcription [58]. Thus, Sp1 is considered as TF obstructing the methylation of CpG islands. However, unavailability of a recent genome-wide analysis of DNA methylation with Sp1 removed makes it impossible to confirm whether Sp1 is necessary for maintaining the non-methylated status in multiple CpG islands.

CTCF is another factor contributing to the maintenance of a non-methylated DNA state. CTCF is identified as TF binding to non-methylated sequences and capable of acting both as transcription activator and repressor. CTCF also acts as insulator; i.e., it blocks enhancer action on promoters and, therefore, is involved in chromatin structure formation [59]. CTCF binds to non-methylated alleles in imprinted loci, disrupting the enhancer-promoter interaction. CTCF binding to the non-methylated maternal allele in the H19/Igf2 locus is not only critical in terms of enhancer-promoter interaction, it also affects the maintenance of the maternal allele in a non-methylated state. Mutations in the CTCF binding sites in this locus resulted in increased methylation of the maternal allele after ovum fertilization, but methylation in the H19/Igf2 locus in germ cells was not disrupted in [60]. CTCF reduction in the oocytes mediated by RNA interference (RNAi) resulted in increased maternal allele methylation in the locus of interest in [61, 62]. Thus, CTCF turns out to be critical in maintaining the maternal allele in the H19/ Igf2 locus in a non-methylated state. CTCF loss in cancer cells leads to hypermathylation in the protein binding sites as well [63]. According to the genome-wide analysis, CTCF is primarily localized in the non-methylated or poorly methylated regions in the stem cells of mice. Nevertheless, some CTCF binding sites are found to be highly methylated [64]. It turns out that methylation intervenes with the CTCF-DNA interaction only at specific positions of the binding site [65]. Mutation in the methylated CTCF binding sites does not cause changes in the methylation level, despite the fact that the presence of CTCF in methylated sequences correlates with lower methylation compared to the regions lacking CTCF recognition sites [66]. Thus, the interaction between CTCF and methylated sequences has nothing to do with maintainance of the methylation level in these regions. It should be noted that DNA methyltransferase knockout cells with a reduced DNA methylation level showed no redistribution of CTCF binding sites onto demethylated regions in [67]. Thus, the DNA methylation, on its own, is not an obstacle to CTCF binding. The sites were found in the imprinted H19/Igf2 locus, which CTCF can bind to in vitro regardless of their methylation level. It is possible that CTCF is not detected on the methylated allele in vivo due to competing binding of methyl-sensitive proteins [68]. Thus, CTCF shows varying DNA binding activity but binding to non-methylated sequences maintains the sequences’ low methylation level.

The search for factors protecting DNA from hypermethylation, akin to CTCF or Sp1, could make it possible to study new mechanisms for maintaining the DNA in a non-methylated state and consider them as targets for manipulating DNA methylation and the transcription activity of genes in conditions associated with DNA methylation abnormalities.

**2.2. Maintaining DNA regions in methylated state **In this chapter, the processes of DNA methylation onset and maintenance are discussed. They are critical to various repeating sequences, imprinted sites, and regulatory elements. De novo DNA methylation involves the DNMT3a and DNMT3b methyltransferases, but, as mentioned above, DNMT1 may manifest de novo activity as well. DNMT3a DNA methyltransferase is responsible for methylation onset in the repeating sequences, regulatory elements, and gene bodies acting as Polycomb protein targets. DNMT3b is critical for methylation onset in the regions of satellite repeats and sequences on inactivated X chromosomes [3, 4]. Histone modifications and interactions with transcription factors are important for DNA methytransferase attraction. Long non-coding RNA and PIWI-interacting non-coding RNA can act as messengers regulating de novo methyltransferase binding to DNA as well [69].

2.2.1. Histone modifications. DNMT3 attraction to DNA is achieved using various mechanisms, including histone modifications. As mentioned above, non-modified H3K4 facilitates the binding of DNA methyltransferases through the ADD domain and amplifies their catalytic activation. In addition, DNA methylation is regulated by H3K36me3/me2 histone modifications. DNMT3 methylates the CpG-rich intragenic sequences of actively transcribed genes in the regions characterized by the presence of H3K36me3-modified histones. DNA binding and DNMT3a-mediated methylation in intergenic regions requires H3K36me2. The PWWP domain of DNMT3 is responsible for the interactions with H3K36me2/ me3 [70, 71].

DNMT3 binding to heterochromatin and repeating sequences is mediated by H3K9 methylation. DNA methyltransferases are attracted to DNA due to interaction with the histone methyltransferases methylating H3K9 (Suv39h1/2, G9a/GLP, Setdb1) and binding to the HP1α and HP1β proteins recognizing methylated H3K9 [72].

2.2.2. Transcription factors attract DNMT to DNA. DNMT3a and DNMT3b are not interchangeable, and their mutations and deletions result in methylation changes in general and specific regions [3, 4]. This has to do with the fact that they are attracted to DNA through interaction with various TFs. As of now, a lot of TFs are being discovered which are capable of interacting with one or both DNA methyltransferases or can be included in a complex with them without interacting directly [73]. Interestingly, these TFs only affect methylation in a limited number of direct targets, which are in many cases restricted to individual target genes. As a result, these TFs may be considered as targets for selective regulation of target gene methylation. Let us discuss some of these factors.

**GCNF**

GCNF (germ cell nuclear factor) participates in methylation onset and maintenance in various promoter regions by directly interacting with DNMT3a/3b methyltransferases [74]. In addition, GCNF may indirectly attract DNMT3 methyltransferases. GCNF in stem cell differentiation binds to the Oct4 promoter and interacts with MBD2 and MBD3, which in turn are included in a single complex with DNMT3. This leads to Oct4 methylation and its transcription suppression in differentiated cells. Since MBD2/MBD3 cannot bind to Oct4 during stem cell differentiation with GCNF knockout, the gene remains active [75]. GCNF ability to regulate Oct4 methylation may be used to analyze a cellular pluripotency status. For instance, GCNF promoter demythilation is observed in somatic cell reprogramming, which enables gene activation during cell differentiation that effectively suppresses Oct4 transcription. These pluripotent cells are mature, but if their reprogramming is not completed, then GCNF promoter methylation is maintained, gene activation does not occur during cell differentiation, and Oct4 remains active in differentiated cells, rendering them potentially oncogenic. Thus, GCNF, or more specifically its promoter methylation, can be considered a maturity marker for pluripotent cells.

**Kaiso (ZBTB33)**

Proteins containing a zinc finger domain often act not only as methyl-DNA-binding proteins, but also as factors contributing to DNA methylation homeostasis [42, 76]. A particular feature of these proteins is their ability to recognize both methylated and non-methylated regions often different in terms of their nucleotide sequences. The zinc finger structure makes it possible to specifically recognize a methylated CG site, most often in a certain context for each TF [77]. The first established proteins to include zinc finger domains interacting with methylated sequences were Kaiso-like proteins: Kaiso (ZBTB33), ZBTB4, and ZBTB38. In addition to zinc fingers, they include the BTB/POZ domain responsible for the protein-protein interaction at their N-end [78 , 79, 80]. Later, other zinc-finger proteins capable of interacting with the methylated DNA were discovered, including Znf57, CTCF, Klf4, Wt1, and Egr1. The strongest affinity to the methylated DNA is demonstrated by Kaiso and Znf57, binding to methylated sequences over 20 times better than to non-methylated sequences. At the same time, the sensitivity to methylated sequences in the remaining zinc-finger proteins is only 1.5-3 times as high or equal to that for non-methylated sequences [81, 82].

Kaiso binds to methylated sequences and regions including CTGCNA [78, 80]. This protein can act as a transcription repressor, with the BTB/POZ domain at the N-end attracting the NcoR and SMRT corepressor complexes, and as transcription activator [83, 84, 85]. Imprinted H19/Igf2 locus is a target for Kaiso that binds to the methylated allele of the locus, and its removal results in ICR1 methylation decrease in the locus [86, 87]. It is possible that methylation decrease following Kaiso removal is due to competition with CTCF, which in turn can bind to methylated sequences and cause their demethylation. In case of Kaiso knockout, methylation decrease is observed in the Oct4 promoter in the embryonic fibroblasts of mice and the TRIM25 promoter in human embryonic renal cells, gene bodies, enhancers, and regions not containing histone modifications [83, 88, 89]. It is shown that TRIM25 promoter demethylation caused by Kaiso removal is reversible by the expression of exogenous Kaiso, which can be included in a complex with DNMT3a/3b [83, 89]. Notably, Kaiso removal in cancer renal cells in humans causes a slight genome-wide methylation increase. This uniform distribution may be associated with the decrease in TET1 dioxygenase transcription; i.e., Kaiso can shift DNA methylation in both directions. Thus, Kaiso not only maintains the required methylation level, but also participates in methylation onset in various loci by interacting with DNA methyltransferases 3a and 3b [89].

The regulating role of Kaiso in DNA methylation may also be associated with its ability to interact with the ubiquitin-like proteins SUMO1,2,3. The SUMO proteins covalently bind to lysine residues in the target proteins, similarly to ubiquitin. Unlike ubiquitination, SUMOylation usually does not cause protein degradation, while affecting cellular localization, activity, and interaction with other factors. Kaiso SUMOylation affects its transcription properties [83]. The presence of six SIM-SUMO interacting motifs in the Kaiso amino acid sequence and non-covalent interaction between Kaiso and SUMO1 allow us to assume that Kaiso can act as a E3 SUMO ligase. SIM sites are sequences of several hydrophobic amino acid residues surrounded by serine or acidic amino acid residues. The so-called non-canonical E3 SUMO ligases include SIM and non-covalently interact with SUMO [90]. Many proteins are SUMOylated in so-called PML and/ or PcG bodies [90, 91]. Kaiso is localized in PcG bodies in the case of exogenous SUMO expression [92]. This allows us to assume that Kaiso not only participates in transcription regulation and DNA methylation maintenance, but may also participate in activity regulation of other factors by affecting their post-translation modifications. For example, SUMOylation of DNA methyltransferases increases their catalytic activity, thereby facilitating an increase in DNA methylation [93]. On the other hand, SUMOylation of the XRC11 excision repair protein is required for effective removal of 5-formyl-and 5-carboxycytosines in stem cell differentiation and subsequently effective DNA demethylation [94]. That is why studying Kaiso in terms of E3 SUMO ligase and searching for its potential targets makes it possible to uncover new activity regulation mechanisms for various factors, including the proteins contributing to DNA methylation.

**Znf57**

Unlike Kaiso, Znf57 contains a KRAB (Krueppel-associated box) domain at the N-end. Znf57 binding to methylated sequences using the KRAB domain attracts the TRIM28 (KAP1) corepressor, which forms a complex with H3K9 histone methyltransferase SETDB1 and DNA methyltransferases DNMT1 (maintenance) and DNMT3a/3b (de novo) [95]. This repressor complex is formed in the transposon region, imprinted loci, and on inactive enhancers [96, 97]. Znf57 removal causes demethylation in imprinted loci and embryonic death [96]. It should be noted that Znf57 is responsible for methylation maintenance, but not onset.

**UHRF1**

UHRF1 plays a key role in DNA methylation maintenance in replication. This explains its expression pattern: UHRF1 is only detected in actively dividing cells (for example, spinal cord cells), where DNA methylation onset in the daughter strand is required in replication, and not detected in terminally differentiated cells (neurons, hepatocytes). UHRF1 binds to methylated and semi-methylated DNA using the SRA domain (SET and RING- associated domain). UHRF1 also includes several domains participating in protein-protein interactions: UBL (ubiquitin-like domain), TTD (tandem tudor domain), PHD (plant homeodomain), and RING (a really interesting new gene domain). These domains ensure interaction with maintenance DNA methyltransferase DNMT1, PCNA, histone deacetylase HDAC1, histone methyltransferases G9a, and SUV39H1, PARP1, etc. [98]. UHRF1 binding to semi-methylated DNA in replication ubiquitinates H3K18 and H3K23 and attracts DNMT1 methyltransferase for methylation establishment in the daughter DNA strand. DNMT1 activity is regulated by interaction with H3K18ub and H3K23ub [99]. In pathogenetic tumor conditions, UHRF1 may also affect methylation onset in the promoters of some genes [100]. UHRF1 removal leads to genome instability, G2/M phase arrest, and apoptosis. The absence of double strand break repairs is observed in cells as well [101]. Thus, UHRF1 contributes to DNA methylation establishment and maintenance.

**MBD proteins**

Methyl-DNA binding proteins with MBD (methyl DNA binding domain) are found among proteins not only recognizing methylated DNA, but also contributing to the binding site methylation. Most MBD proteins are involved in the formation and functioning of the nervous system. There are only four factors in this family (MBD1, MBD2, MBD4, and MeCP2) capable of binding to methylated DNA. These MBD proteins show the strongest affinity to methylated CpG islands [102]. In most cases, these proteins act as interpreters of methylation: i.e. they attract corepressors or compete with transcription activators for DNA binding. However, some recent studies show that these factors can also contribute to DNA methylation establishment and maintenance. It was shown that MeCP2 knockout leads to the occurrence of both hypo- and hypermethylated regions in various types of neurons in mice [103]. The mechanism behind the effect of MBD proteins on the methylation level is yet to be studied. MBD1 regulates methylation in the promoters of the Htr2c serotonin receptor gene and bFGF growth factor [104, 105]. MBD1 knockout leads to Htr2c reactivation, which is considered among the causes of deviations in hippocampal neurogenesis, learning disorders, and occurrence of autism symptoms associated with social behavioral changes, attention deficit, and serotonin activation abnormalities in gene-knockout animals [104]. Reactivation of the bFGF growth factor in the case of MBD1 knockout affects the ability to maintain the pluripotent state of stem cells, whose regulation is important for the subsequent differentiation into cells of the nervous system [105].

Thus, there are factors, such as UHRF1, that contribute to genome-wide methylation maintenance and ones (MBD, Kaiso, and GCNF proteins) that regulate methylation in a specific variety of targets. The latter are of special interest, since identification of their binding sites, whose methylation level is affected by the inactivation or mutations of these factors, could make it possible to manipulate the methylation levels in their targets by changing their activity. Interestingly, the desired changes in DNA methylation may also be regulated by activity modulation of DNA methyltransferase using post-translation modifications: Kaiso is a potential E3 SUMO ligase.

## 3. DNA METHYLATION EDITING

Using advanced DNA editing methods to change methylation levels in certain regions is one of the ways used to alter their transcription activity. This approach makes it possible to alter promoter and enhancer activity using mutated dCas9 endonuclease incapable of DNA cutting. To ensure DNA hypermethylation, dCas9 is bound to the catalytic domain of DNMT3, whose methyltransferase activity is targeted on the region of interest [106]. The TALEN and zinc finger domain may be used instead of dCas9, but the editing system based around dCas9 remains the most accessible one. The main problems with this editing technique are as follows: 1) the methylation level is not high enough, and 2) DNA demethylation occurs after a certain number of cell divisions. To solve these problems, DNMT3L acting as a cofactor amplifying DNA methylation is added to the catalytic domain of DNMT3. A high methylation level is maintained during a lasting cell division by introducing the chimeric construct dCas9-Ezh2 or dCas9-KRAB into the cells. Ezh2 trimethylates H3K27, and the KRAB domain of Znf57 acts as a base that can be used to assemble a repressor complex that modifies histones and methylates DNA [107]. It is also necessary to identify which factor – Ezh2 or KRAB – would be more effective in suppressing the transcription activity in the region of interest [107].

The key advantage of DNA methylation editing, compared to DNA editing, is that the nucleotide sequence remains intact while only DNA modification changes. These changes are reversible, and almost any sequence in the genome can be edited.

## 4. DNA METHYLATION AND PATHOGENETIC CONDITIONS

In recent years, the relationship between the regulatory mechanism of DNA methylation and various pathogenetic conditions, especially oncogenesis, rheumatoid arthritis development, and various neurological diseases, has been uncovered [11, 111]. Two categories of clinical significance of the changes in the DNA methylation level can be identified. The first one includes the cases where DNA methylation may act as a marker for a developing pathogenetic condition. The second one includes cases where changes in DNA methylation and the activity of methyl-DNA binding proteins affect the course and progression of the condition.

**4.1. DNA methylation as a diagnostic and predictive marker of disease progression**

The DNA regions whose methylation changes can be detected in damaged organs or tissues, blood genomic DNA, DNA from various body fluids, and circulating-free DNA are selected as markers of disease progression. Markers making it possible to quite accurately predict oncological diseases at their early stages, evaluate the effect of therapy, detect recurrent cases, and even identify tumor types in some cases, have been selected [112, 113, 114].

**4.2. DNA methylation as a target for therapy in various pathogenetic conditions**

Hypermethylation in the CpG islands located in suppressor gene promoters, leading to their inactivation, is often detected during oncogenesis. Tumor suppressor genes can be activated, albeit inconsistently, through promoter demethylation. For instance, 5-azacitidine reducing DNA methylation is used as an active substance in decitabine used as therapy in acute myeloid leukemia and myelodysplastic syndrome. However, instead of targeting a specific gene, this drug affects the whole genome, causing its instability and damaging the DNA, which may have severe consequences for the patient [115]. Methylation in the promoters of tumor suppressor genes may be reduced by inactivation of the catalytic activity of the maintenance DNA methyltransferase. Inhibitors of DNMT1 DNA methyltransferase RG108 and SG102 are less toxic than 5-azacitidine. They do not change methylation in satellite repeats but affect promoter demethylation, including in some suppressor genes [116, 117]. The key limitation of these inhibitors is the small quantity of targets; i.e., the regulatory elements of suppressor genes. The catalytic activity of DNMT1 may also be suppressed using oligonucleotides that form quadruplex structures [53]. Attempts are made to manipulate DNA methylation using the editing system. The bottleneck of this approach is the delivery of dCas9 or its analogues to target organs and tissues [118]. Hepatocytes, where the editing system can be delivered via injection (for example, tail vein injection in mice), are one of the most accessible targets. Attempts to reduce methylation in the Fgf21 promoter in the liver of mice have been described. Fgf21 codes for the factor participating in glucose and cholesterol metabolism. Introduction of dCas9 with the catalytic domain of TET1 resulted in a short-term methylation decrease in the promoter on the sixth day after injection, and as early as the 14^th^ day the methylation level was restored in [119]. Thus, stable DNA methylation editing in a living organism is yet to be achieved.

**4.3. Methyl-DNA binding proteins as new targets for therapy**

When selecting a therapy target, one should take into consideration how critical is the inactivation of a factor to the organism. Knockout or mutations in the methyl-DNA binding proteins MBD1, MBD2, MeCP2, and Kaiso result primarily in behavioral deviations not disrupting vital processes, which may be reversed upon restoration of protein expression as in the case of MeCP2 [120, 121]. Inactivation of these proteins changes the general methylation level insignificantly and does not lead to genome instability and reactivation of repeating elements. Hence, the MBD proteins Kaiso and their homologue ZBTB4 enjoy an advantage as potential targets. The search for the target genes of these factors associated with pathogenetic conditions seems a promising line of research.

**Fig. 5:**
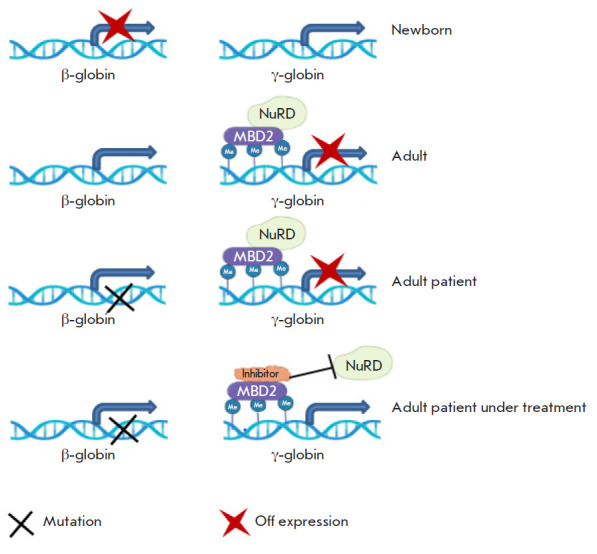
Model of the functional significance of the interaction between the MBD2 protein and the NuRD repression complex in the regulation of the gamma globin gene in beta-telassemia [122, 123]

For instance, investigation of the binding sites in methyl-DNA binding proteins made it possible to identify the gamma globin gene as a methyl-dependent target. A gradual transition of hemoglobin types occurs during the human organism’s development: the epsilon globin gene is transcribed in the embryonic period; gamma globin – at birth; and beta globin – in adulthood. Patients with the sickle-cell disease and beta thalassemia show an abnormal expression of or mutations in the beta globin gene, leading to severe consequences. Reactivation of the normal form of gamma globin would make it possible to restore a normal hemoglobin level in the blood. The methyl-DNA binding protein MBD2 regulates the attraction of the NuRD corepressor complex to the promoter of the gamma globin gene in blood cells and maintains it in an inactive state in adults [122]. MBD2 removal leads to a 20-fold increase in the expression of the gamma globin gene [123]. Transcription of the gamma globin gene may be activated by disrupting MBD2 binding to the NuRD corepressor complex and its components using inhibitors (Fig. 5). Various models have shown that exclusive inactivation of MBD2 does not affect the body function. MBD2-knockout mice demonstrate disrupted maternal behavior while nurturing and feeding their offspring [120, 124]. Aside from this, MBD2 removal does not cause any pronounced neurological deviations. Therefore, we can expect MBD2 inhibition to not cause severe side-effects in humans. Thus, the methyl-DNA binding repressor activity of MBD2 may be used for hemoglobin level restoration in patients with sickle-cell disease and beta thalassemia. However, inactivating the methyl-DNA binding protein case cited above is not always necessary. For instance, mutations in or inactivation of the methyl-DNA binding protein MeCP2 lead to Rett syndrome development. MeCP2 knockout in mice, similarly to mutations in this gene in humans, causes neurological changes. Notably, changes occurring in nerve cells due to MeCP2 removal or mutation are reversible [125]. The MeCP2 mutations identified in patients with Rett syndrome include, among others, point mutations causing MeCP2 degradation but not affecting the structure of its DNA-binding and repressor domains [126]. When stabilized, this protein can still fulfill its functions [127]. A search for small molecules binding to MeCP2 ubiquitination sites could make it possible to prevent its ubiquitination, with subsequent degradation, and restore the protein’s functional activity.

Thus, the search for and characterization of the binding sites in methyl-DNA binding proteins are necessary for the identification of potential targets whose activity is regulated by DNA methylation and the formation of repressor complexes. Further analysis of the various pathogenetic conditions associated with the target genes of methyl-DNA binding proteins allows us to consider methyl-DNA binding proteins as targets for therapy, while investigation of the mutations in methyl-DNA binding proteins makes it possible to understand when functional changes caused by mutations can be compensated, and when that is impossible.

## CONCLUSIONS

DNA methylation is a regulatory element critical to gene expression, genome stabilization, inactivation of repeating sequences, establishment of imprinting, and X-inactivation. Advanced genome-wide sequencing methods allowed us to determine the DNA methylation pattern across the whole genome, including various repeating sequences. It opened new opportunities in terms of the identification and characterization of regulatory elements whose activity may be disrupted by various pathogenetic conditions. As of now, a lot of TFs participating in methylation onset and maintenance, demethylation, or interpretation of methylated DNA have been discovered. Methylation can facilitate TF attraction or interfere with it; i.e., the DNA methylation level affects selection of the protein factors interacting with DNA and alternating between attraction of transcription activators and repressors. Discovery of new DNA methylation-dependent factors and investigation of the activating and repressor complexes they are included in allow us to consider these factors as new therapy targets to be manipulated to achieve a more nuanced effect compared to genome-wide inhibition of DNA methylation. Thus, the study of new methyl-DNA sensitive proteins could make it possible to identify new approaches and therapeutic targets for the management of various pathogenetic conditions associated with DNA methylation onset and regulatory changes.

## Abbreviations

TF: transcription factors
